# Peer‐Mentor Support for Older, Vulnerable Patients With Ischemic Heart Disease: A Mixed Methods Process‐Outcome Evaluation

**DOI:** 10.1111/jan.16899

**Published:** 2025-03-17

**Authors:** Maria Pedersen, Rikke Agnete Petersen, Takyiwa Boateng, Ingrid Egerod, Dorthe Overgaard, Birgitte Bøcher Bennich

**Affiliations:** ^1^ Department of Nursing and Nutrition University College Copenhagen Copenhagen Denmark; ^2^ Department of Intensive Care University of Copenhagen, Rigshospitalet Copenhagen Denmark

**Keywords:** coronary heart disease, mentors, mixed methods design, nursing, older people, rehabilitation

## Abstract

**Aim:**

To achieve a deeper understanding of the results of a primary randomised controlled trial to clarify the potential effective mechanisms and barriers of a peer‐mentor intervention.

**Design:**

Mixed methods process‐outcome evaluation of the intervention.

**Method:**

Qualitative and quantitative data were collected during the intervention in a during‐trial set‐up, that is, a convergent design.

**Results:**

The qualitative and quantitative findings mostly confirmed and expanded each other, identifying several mechanisms that facilitate the effectiveness of peer‐mentor support during cardiac rehabilitation, such as mentors' experience‐based knowledge and motivation. However, barriers related to lifestyle changes among older, vulnerable patients (e.g., mentee concerns about heart‐healthy diets) and psychological outcomes (e.g., mentees' resilience) may minimise the effectiveness.

**Conclusion:**

Peer‐mentoring holds potential for supporting older, vulnerable patients during cardiac rehabilitation. However, ensuring that peer‐mentors are well‐suited for their role and capable of providing motivational, experience‐based support is crucial, as is the need for tailored mentorship and consideration of specific patient populations needing mentor‐supported cardiac rehabilitation.

**Implications and Impact:**

Cardiac rehabilitation faces challenges due to high drop‐out rates, particularly among older individuals, females, and vulnerable patients. Peer mentoring, a low‐cost intervention, holds promise for supporting these groups in cardiac rehabilitation programmes.

**Reporting Method:**

The study adheres to the ‘Systematic Development of Standards for Mixed Methods Reporting in Rehabilitation Health Sciences Research’, ‘Good Reporting of A Mixed Methods Study’ and ‘Template for Intervention Description and Replication’.

**Patient and Public Contribution:**

A group of patients with cardiovascular disease actively contributed to developing and implementing the intervention.

**Trial and Protocol Registration:**

ClinicalTrials.gov Identifier: NCT04945486—prospectively registered before the first participant was recruited

**Summary**

- What does this paper contribute to the wider global clinical community?
  ○ Peer mentors are valuable collaborators that healthcare professionals should engage with to enable the rehabilitation of older, vulnerable patients.

## 1 Introduction

Mortality rates related to ischemic heart disease (IHD) are on a downward trend (Hammond‐Haley et al. 2021). Consequently, there is a growing population of IHD patients who require subsequent cardiac rehabilitation (CR). Studies have demonstrated the effectiveness of CR in reducing hospital admissions, cardiovascular mortality, and enhancing the quality of life for IHD patients. However, non‐attendance and drop‐out rates from CR programmes pose significant challenges, especially among older individuals, females, and vulnerable patients (Pedersen et al. 2018a; Ruano‐Ravina et al. 2016).

Vulnerability in this context includes patients with a non‐Western background, low socioeconomic position (SEP) characterised by low educational levels, and those living alone (Pedersen et al. 2018b; Ruano‐Ravina et al. 2016). Feasible and effective interventions aimed at increasing CR attendance for older, vulnerable groups are therefore warranted.

## 2 Background

Peer‐mentoring (i.e., mentoring by a person with a similar life situation or health problem) is a low‐cost intervention, which has proven feasible in older, vulnerable patients with IHD (Pedersen et al. 2022). Peer‐mentoring is effective in improving physical and psychological outcomes in older patients (Dorgo et al. 2013; Goldman et al. 2013; Pfeiffer et al. 2011), and in enhancing self‐efficacy and benefit health and well‐being among patients with cardiovascular disease (Parry and Watt‐Watson 2010).

The present mixed methods process‐outcome evaluation is an ancillary study to a primary randomised controlled trial (RCT) and part of the ongoing research programme ‘Heartened’ (in Danish: ‘HjertensGlad’), developing and testing a peer‐mentor intervention among older, vulnerable patients with IHD (Pedersen et al. 2022).

While randomised controlled trials (RCTs) are commonly regarded as the benchmark for evaluating intervention efficacy, various forms of process evaluation play an important role in providing insight into causal mechanisms (Moore et al. 2015). Process evaluation facilitates understanding of intervention implementation practices and the identification of contextual factors influencing their effectiveness. When RCT outcomes yield null results, qualitative data can indicate reasons for the intervention's ineffectiveness, thereby reinforcing future study design improvements. Conversely, in cases of positive RCT outcomes, qualitative data can help pinpoint effective mechanisms (O'cathain 2018). Thus, a mixed methods process‐outcome evaluation can help to distinguish between genuinely ineffective interventions and interventions that might be effective, if they were better implemented or implemented in a different context (O'cathain 2018). Thus, process evaluation can be employed to continually refine an underpinning program theory that describes how the intervention is expected to lead to its effects and under which conditions (Skivington et al. 2021).

The results of the primary RCT study showed a significant positive effect of the peer‐mentor intervention related to CR attendance, that is, mentor support significantly increased initial attendance in the first CR session and long‐term CR adherence (Pedersen et al. 2024). The primary RCT study, however, found no statistically significant effect on reported symptoms of anxiety or depression, and no statistically significant differences in health‐related quality of life, dietary outcomes, mean scores relating to self‐efficacy, or in the proportion of persons who reported being physically active between the intervention and control group (Pedersen et al. 2024).

The aim of this study was to achieve a deeper understanding of the results of the primary RCT to clarify potentially effective mechanisms and barriers of the intervention.

## 3 Methods

**FIGURE 1:**
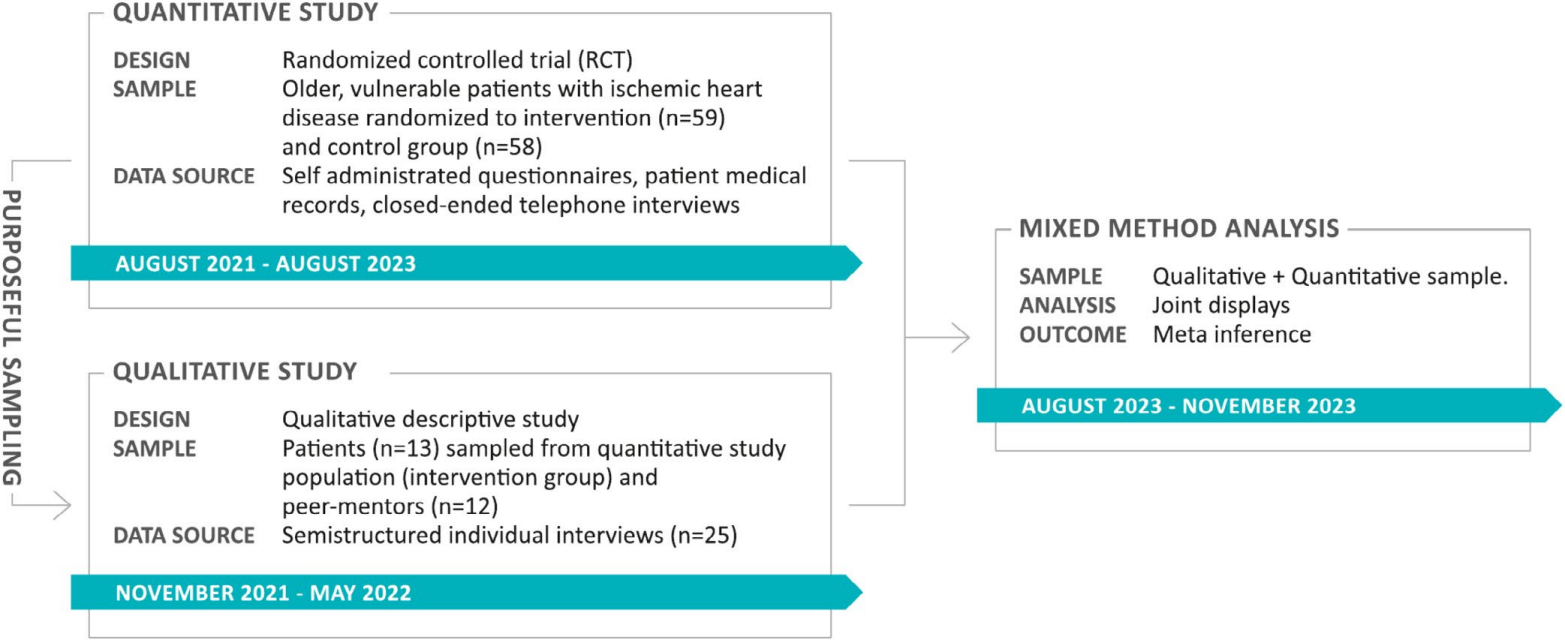
Mixed methods study design.

The study is designed as a mixed methods process‐outcome evaluation (O'cathain 2018). The study adheres to the EQUATOR guidelines ‘Systematic Development of Standards for Mixed Methods Reporting in Rehabilitation Health Sciences Research’ (MMR‐RHS) (Tovin and Wormley 2023) and the principles of ‘Good Reporting of A Mixed Methods Study’ (GRAMMS) (O'Cathain et al. 2008). The intervention is reported according to the ‘Template for Intervention Description and Replication’ (TIDieR) checklist (Hoffmann et al. 2014). The qualitative data were collected during the RCT in a during‐trial set‐up that is, a convergent design, where qualitative and quantitative data are collected concurrently (Creswell and Plano Clark 2018; O'cathain 2018). The rationale for employing this mixed methods design was to gain a comprehensive understanding of effectiveness of peer‐support. Equal priority was given to both qualitative and quantitative data. The qualitative data were collected and analysed prior to the RCT results being known, subsequently the qualitative data were used to explain the primary RCT results in an iterative process, illustrated in Figure 1.

## 4 Intervention

The intervention was administered by peer‐mentors who met specific criteria. These peer‐mentors were individuals diagnosed with myocardial infarction (MI) or a similar condition, such as percutaneous coronary intervention/coronary artery bypass grafting or chronic ischaemic heart disease. Additionally, they had previously participated in a CR program, serving as positive role models. The peer‐mentor intervention was developed based on a pre‐established program theory, also known as a logic model (Skivington et al. 2021). This model outlines how the intervention is expected to produce its effects and the circumstances under which these effects are expected to occur (Skivington et al. 2021). The program theory was based on the concept of self‐efficacy (Bandura 1997), enabling a theoretical understanding of the likely process of effect (Skivington et al. 2021). General self‐efficacy pertains to an individual's convictions regarding their overall capacity to accomplish tasks and attain desired objectives, such as participating in CR and effecting lifestyle modifications. These beliefs in self‐efficacy are synthesised from four primary sources of information: (1) Mastery experiences, (2) Vicarious experiences, (3) Verbal persuasion, and (4) Physiological and affective states (Bandura 1997). Vicarious experiences have the potential to positively alter self‐efficacy beliefs through the transmission of competencies by comparison to similar others (e.g., a peer‐mentor) and thus constitute a fundamental mechanism underpinning the presumed beneficial impact of mentor interventions.

**FIGURE 2:**
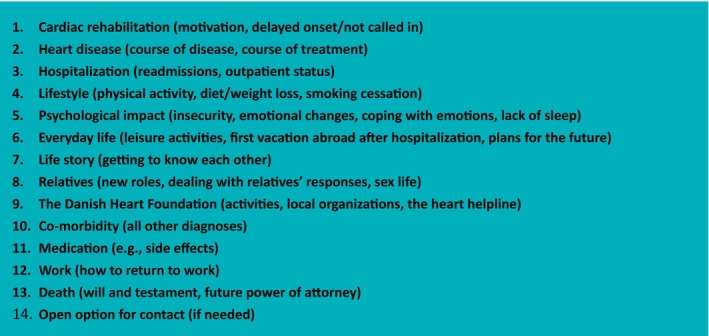
Conversation points during mentor‐mentee interactions.

A peer‐mentor (IHD survivor who successfully attended CR) was matched with an older, vulnerable mentee (recent IHD survivor) according to the mentee's personal preferences, such as age, gender, and disease trajectory. Mentors provided support and guidance to their mentees during the 24‐week intervention period, drawing on their first‐hand illness experience. The frequency of mentor‐mentee interactions varied widely, averaging about five contacts. Some mentees required only one interaction, while others had as many as 14 meetings. These contacts occurred through various interactions, including face‐to‐face meetings or telephone conversations. The contacts lasted from minutes to several hours, depending on mentee needs. Most face‐to‐face meetings occurred in the mentee's homes, while some chose to meet in public places. Peer mentors were volunteers offering support based on their disease experiences. They participated in a two‐day online training course organised by researchers and a psychologist, focusing on relationship‐building, communication skills, and understanding psychological reactions to IHD and lifestyle changes. Training materials can be obtained by contacting the main author. After training, the content of mentor‐mentee meetings was minimally regulated. Mentors were trained to address the themes most relevant to the mentees. Figure 2 provides an overview of the dominant discussion topics in these interactions. Hence, interactions were tailored by mentors to meet the specific needs of their mentees. Additionally, peer mentors received bimonthly supervision from the research team and psychologist. The intervention and program theory are described in greater detail elsewhere (Pedersen et al. 2022, 2023).

## 5 Quantitative Data Source

From August 2021 to March 2023, a research nurse assessed the eligibility of 1620 patients admitted to a university hospital in Denmark. Among these, 117 patients met the mentee criteria and were enrolled in the primary RCT. This trial aimed to evaluate whether a 24‐week peer‐mentor intervention could enhance CR attendance and subsequently improve lifestyle (diet and physical activity) and psychological outcomes (self‐efficacy, anxiety, depression, quality of life) among older vulnerable patients diagnosed with IHD. All 117 patients from the RCT are included in the quantitative analysis in this paper, with 59 patients enrolled in the intervention group and 58 in the control group.

Detailed inclusion and exclusion criteria are provided in Table 1.

**TABLE 1:** Inclusion and exclusion criteria.

Primary inclusion criteria (all mandatory)	≥ 65 years, *AND* diagnosed with IHD, *AND* referred to cardiac rehabilitation.
Secondary inclusion criteria (one mandatory)	Female, OR low socioeconomic position^a^, OR lone dwelling, OR nonwestern background^b^
Exclusion criteria	Patients unable to provide written consent.

^a^ Defined as vocational educational level or below.

^b^ Defined as persons not born in one of the 27 EU countries, Andorra, Iceland, Liechtenstein, Monaco, Norway, San Marino, Switzerland, Great Britain, Vatican City, Canada, the USA, Australia, or New Zealand.

Quantitative outcomes were CR‐attendance (initial attendance in the first CR session and long‐term CR adherence), self‐efficacy, symptoms of anxiety and depression, quality of life (QoL), dietary quality, and physical activity. Outcomes were assessed at three time‐points: T0 Baseline, T12 (12‐weeks) and T24 (24‐weeks). Self‐reported CR attendance was assessed via closed‐ended telephone interviews. This is a validated method of assessing CR attendance, with a Cohen's kappa of 0.846 (Kayaniyil et al. 2009). Self‐efficacy was measured using the validated general self‐efficacy scale (GSE) (Schwarzer and Jerusalem 1995). Higher GSE scores indicate more positive outcomes, and the scale has a high reliability (Cronbach's alpha = 0.94) (Luszczynska et al. 2005). Symptoms of anxiety and depression were measured using the validated hospital anxiety and depression scale (HADS) (Zigmond and Snaith 1983). Lower HADS scores indicate a positive outcome. The scale has good reliability, with Cronbach's alpha values of 0.87 for anxiety symptoms and 0.82 for depression symptoms (Christensen et al. 2020). QoL was measured using the validated HeartQoL questionnaire (Oldridge et al. 2014). Higher HeartQoL scores indicate more positive outcomes, and the scale has good reliability (Cronbach's alpha = 0.87) (Oldridge et al. 2014). Dietary quality and physical activity were measured using the validated ‘HeartDiet’ questionnaire, thus dietary data were captured using the 19‐item food‐frequency questionnaire, consisting of two subscales: a fish‐fruit‐vegetable score (range 0–100); and a fat score (range 0–100). A minimum of 75 points had to be achieved on both subscales for a diet to be categorised as ‘heart‐healthy’ (Kristensen et al. 2024). Higher scores indicate more positive outcomes, with a statistically significant correlation between the HeartDiet questionnaire and other reliable food frequency questionnaires (Laursen et al. 2018). In the primary RCT study, the cut‐off for being physically active was physical activity > 5 times/week. In the present study, we exploratively used a higher cut‐off (> 6 times/week) to comply with the Danish Health Authority recommendations (Gelius et al. 2020).

Finally, mentees were asked about their satisfaction with the mentor‐intervention and self‐reported overall estimated effect of the peer‐mentor intervention in self‐administered questionnaires. Peer‐mentors kept a detailed record of conversation topics during mentor‐mentee interactions to assess the content of the delivered intervention.

## 6 Qualitative Data Source

Qualitative data were gathered from a purposefully selected sample comprising 13 mentees and 12 peer mentors, all drawn from the RCT population between T12 and T24, enabling experience with peer‐mentor support without the final outcome being known. Semi‐structured individual interviews were conducted by the last author between November 2021 and May 2022. The last author, with a nursing background and proficiency in qualitative methods, had no prior affiliations with peer‐mentors or mentees. An interview guide, exploring the mentor‐mentee relations and aligning with themes related to the quantitative variables in the RCT, was employed during interviews. The interviews were audio‐recorded and transcribed verbatim.

## 7 Quantitative Analysis

Quantitative data are presented as frequencies, percentages, means, and standard deviations depending on whether the data were normally distributed. All quantitative analyses presented in this paper represent explorative ancillary findings that are not shown in the RCT paper (Pedersen et al. 2024). Quantitative analyses were carried out in IBM SPSS statistics (Statistical Package for the Social Sciences, version 25) by the second author.

## 8 Qualitative Analysis

The qualitative interview data were initially analysed by the first author, the second author, and the last author, all with a background in nursing, using the six stages of thematic analysis suggested by Braun and Clarke (Braun and Clarke 2006): Data familiarisation, creating initial codes, developing, refining, and defining themes and sub‐themes, and crafting analytical narratives with quotes illustrating the themes and sub‐themes for use in mixed methods analysis. All authors participated in discussion of the final analysis. NVivo (version R1.6) was used to support the organisation of data. Subsequently, the reporting of the various themes was segmented based on quantitative variables to facilitate the following mixed methods analysis.

## 9 Mixed Methods Analysis

In the mixed methods analysis, quantitative and qualitative results were integrated using joint displays—a visual format that merges both types of findings. This approach, as advocated by Guetterman, Fetters, and Creswell (Guetterman et al. 2015), allowed for a more comprehensive understanding of the results derived from the primary RCT. The qualitative analysis was used to sum up analytical narratives with illustrative quotes; these narratives, along with themes and sub‐themes, were then incorporated into the joint displays alongside descriptive quantitative data. Through mixed methods inferences, or meta‐inferences, the alignment between qualitative and quantitative findings was assessed. This evaluation resulted in three possible outcomes: confirmation, discordance, or expansion, indicating whether qualitative and quantitative data reinforced, contradicted, or enhanced insights related to barriers or mechanisms of the primary RCT outcomes, clarifying potential mechanisms altering mentees' self‐efficacy beliefs (Guetterman et al. 2015).

## 10 Findings

The first findings presented here are not part of the joint displays. Figure 2 describes the 14 conversation topics recorded by the mentors during the peer‐mentor interactions in a non‐prioritised sequence. The topics suggest potential avenues for changes in the mentee's self‐efficacy beliefs.

**FIGURE 3:**
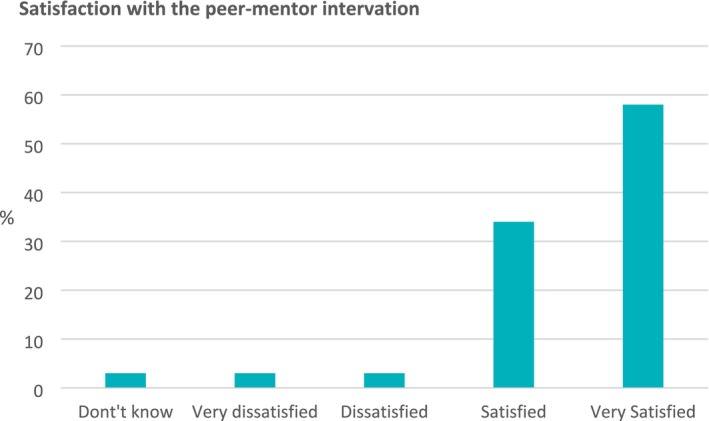
Mentee satisfaction with peer‐mentor intervention.

The bar chart (*n* = 59) in Figure 3 shows that most of the mentees were satisfied (34%) or very satisfied (58%) with the peer‐mentor intervention.

**FIGURE 4:**
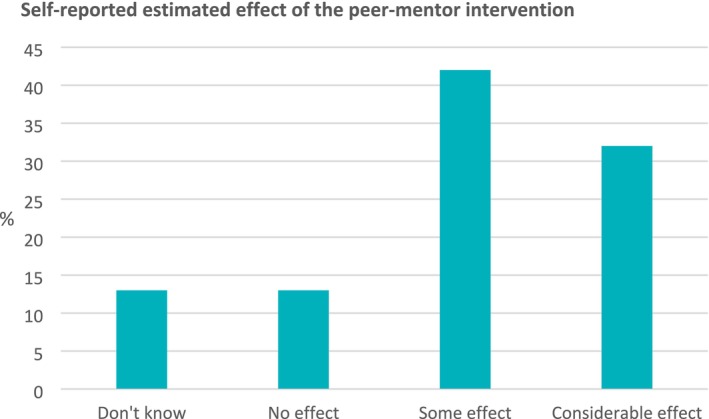
Mentee experienced the effect of the peer‐mentor intervention.

The bar chart (*n* = 59) in Figure 4 suggests that most of the mentees experienced a positive overall effect of the peer‐mentor intervention, as 42% experienced some effect and 32% a considerable effect.

Table 2 shows the analytical steps during thematic analysis, including examples of quotes supporting sub‐themes and themes used in the joint displays.

**TABLE 2:** Example of analytical steps in the thematic analysis.

Quotes	Sub‐themes	Themes
M1: If you haven't been offered cardiac rehab, you should ask for it. It might sound tedious, but it's one of the best things you can do. You get to meet other people like me during exercise and physiotherapy, and they (the professionals) have a good grip on you and make sure you don't overdo it P4: At home, I follow the Heart Foundation's program, and it's more effective than if I had to drive all the way to [the offered CR location]	Mentoring drives rehabilitation Convenient self‐rehabilitation	Rehabilitation participation depends on convenience and encouragement from mentor
P11: Rather small steps I can keep without falling back … nothing dramatic P3: We are simply getting older, so it can go wrong whether you eat vegetables every day or not P2: My husband is very supportive about diet. We have totally changed our diet and I have also lost six or seven kilograms P23: I could see that he (mentor) was doing well. They (CR attendants) have taken charge of their life again, they have moved on and have been to CR many times, and they are doing well P23: I think what he (mentor) realised that he really just needed to push me a little bit to get me to start training	Avoiding dietary relapse Aging shapes dietary perspectives Family support for sustainable lifestyle changes Role model for activity Duality of physical engagement and constraints	Lifestyle changes rely on meaningfulness, role models and family support
P14: Then it's just getting back on the horse. But I feel the sadness—that you lose the will to live. That's why you have to be careful not to withdraw, and I've been able to talk to my mentor about that P11: the mentor can help someone who is a bit panicked or, to put it bluntly, scared to death, to trust their heart P23: Your living conditions shape you… I've almost been like a cork. You can also pull it down under water, but if you let go, it just pops back up	Restoring trust in the heart Life experiences build resilience	Resilience and self‐efficacy come with life experience

### 10.1 Cardiac Rehabilitation Attendance

**FIGURE 5:**
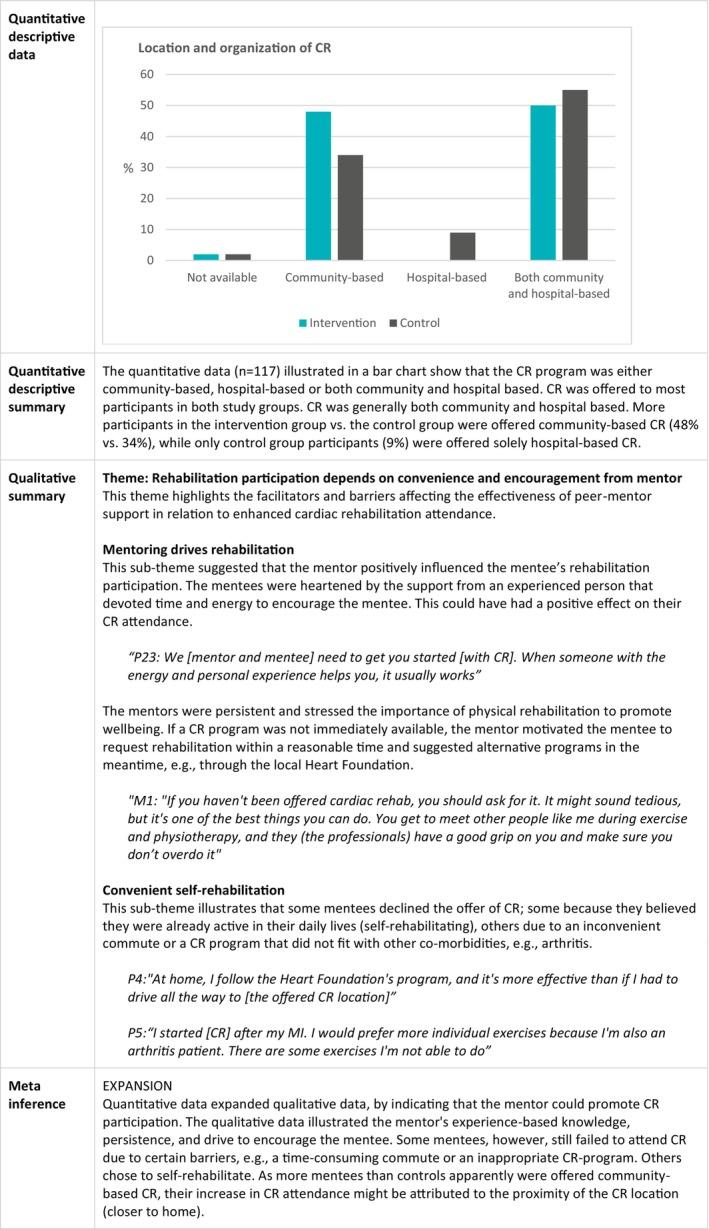
Joint display of cardiac rehabilitation attendance.

The joint display in Figure 5 shows that the qualitative and quantitative findings regarding CR attendance expand each other. The qualitative data illustrate the criticality of the mentor's experienced‐based knowledge, persistence, and motivation in encouraging the mentee to attend CR. The qualitative data, however, reveal obstacles such as long commutes or perceived physical inadequacy, leading mentees to choose self‐rehabilitation at home. The quantitative descriptive data show a disparity in CR availability between the two study groups, as more mentees were offered community‐based CR than controls. Hence, a plausible mechanism of the intervention groups' improvements in CR attendance could partly be attributed to an easier commute, as travel time and proximity to home affected attendance.

### 10.2 Dietary Improvement

**FIGURE 6:**
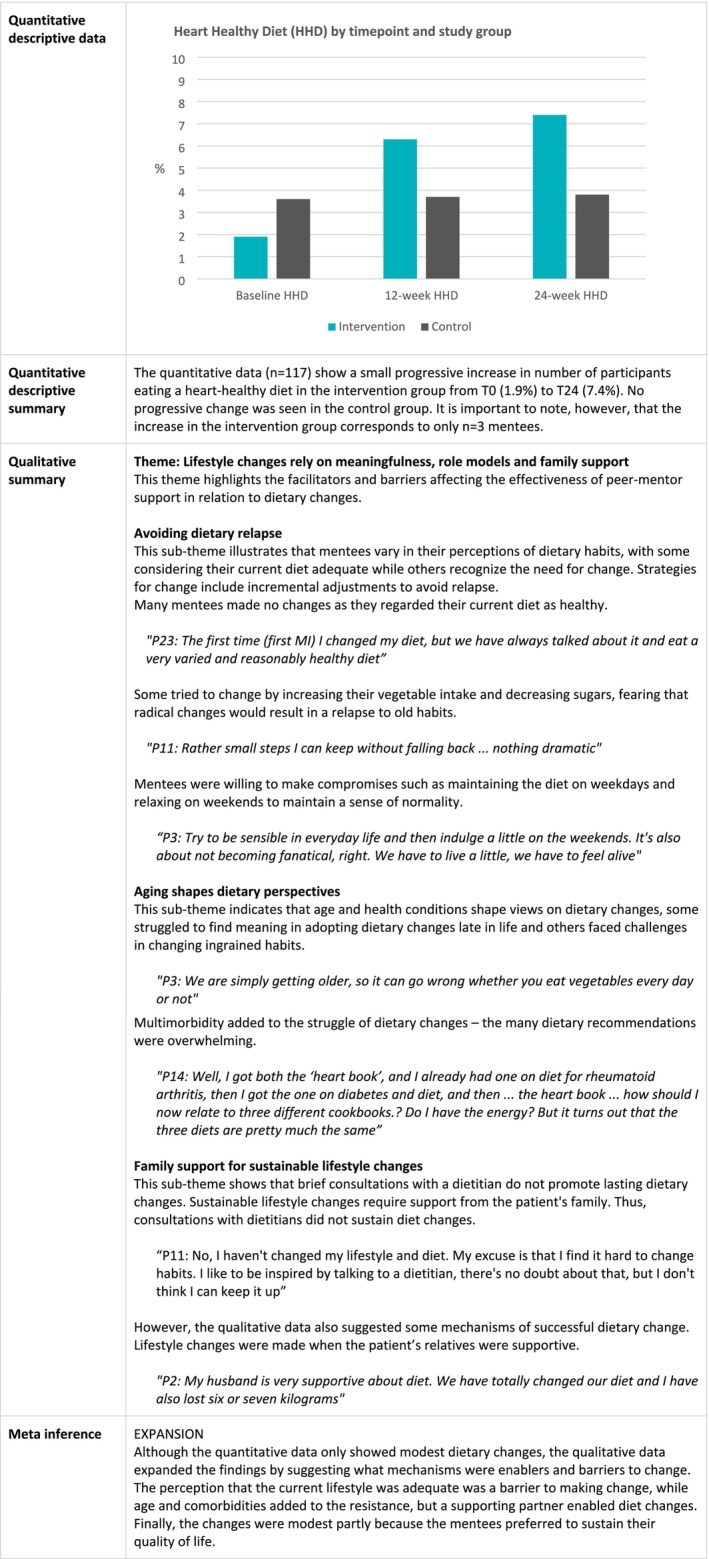
Joint display of changes in dietary quality.

Figure 6 joint display shows that the qualitative and quantitative data regarding dietary changes expanded each other. Qualitative insights complemented the quantitative data concerning the lack of major dietary modifications during the trial. The qualitative analysis suggested various mechanisms that hinder dietary improvements and limit self‐efficacy in relation to dietary changes. Firstly, some mentees perceived themselves as already having a healthy lifestyle, thus saw little need for dietary guidance. Secondly, age and comorbidities altered perspectives and challenged dietary changes. Thirdly, having a supportive partner emerged as pivotal for sustaining dietary modifications. Lastly, mentees prioritised actions that enhanced their quality of life and were reluctant to adopt drastic dietary alterations due to the potential negative impact on their quality of life. Consequently, adopting a less rigid stance towards dietary changes appeared to be a preferred strategy among mentees.

### 10.3 Physical Activity Improvement

**FIGURE 7:**
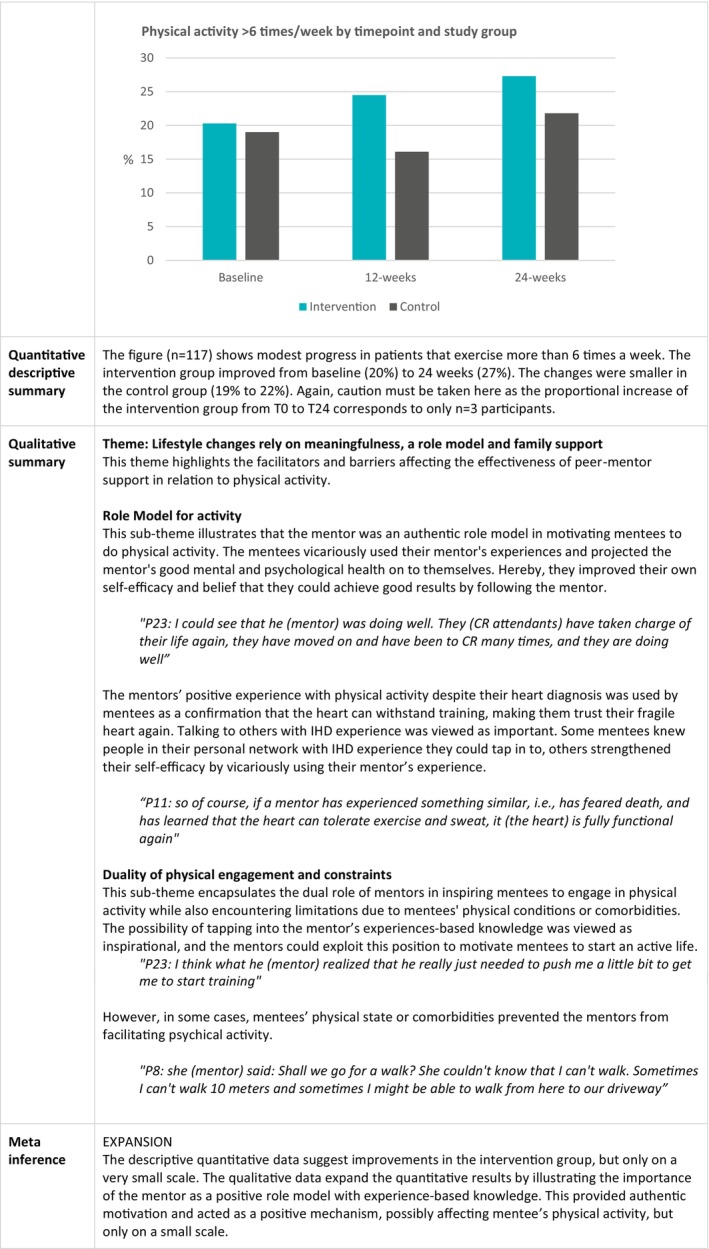
Joint display of changes in physical activity.

The joint display in Figure 7 shows that qualitative and quantitative data describing physical activity expand each other. The current data suggest improvements in the intervention group, but only on a small scale. The qualitative data expand quantitative knowledge by illustrating the importance of the mentor being a positive role model with experience‐based knowledge. This can provide authentic motivation and improve self‐efficacy as a positive mechanism, possibly affecting the mentee's physical activity on a small scale.

### 10.4 Changes in Anxiety and Depression

**FIGURE 8:**
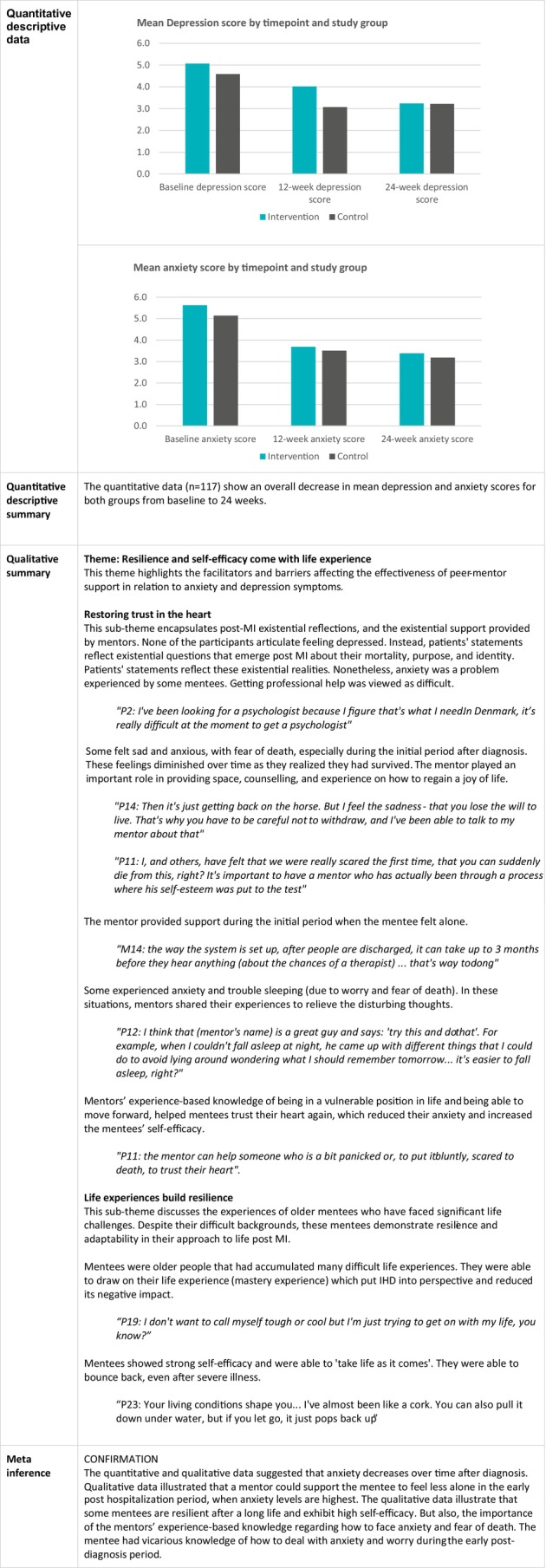
Joint display of changes in depression and anxiety.

The joint display in Figure 8 shows that the qualitative and quantitative data regarding anxiety and depression confirm each other. Both quantitative descriptive data and qualitative analyses indicate a reduction in anxiety levels over time following diagnosis. Qualitative findings highlight the significant role of a mentor in providing support during the critical early post‐hospitalisation phase, when anxiety levels are typically elevated. The mentor serves as a valuable source of vicarious experience, helping to alleviate anxiety and worry during the initial post‐diagnosis period. However, the qualitative data underscore the resilience displayed by some mentees who have lived through considerable life experiences, showcasing high levels of self‐efficacy, possibly a mechanism reducing the long‐term effect of mentor support on anxiety and depression symptoms.

## 11 Discussion

This mixed methods process‐outcome evaluation enabled a deeper understanding of the results in the primary RCT by suggesting potential effective mechanisms and barriers of the mentor intervention. Qualitative and quantitative data mostly confirmed and expanded each other, indicating that mentees overall experienced mentor support as positive. Previous studies have demonstrated that a peer‐mentor might positively affect CR attendance (Carroll et al. 2007; Pedersen et al. 2022) and the data affirmed the mentor's important role in encouraging CR attendance. Several mechanisms might explain the positive effect; firstly, the mentor's experience‐based knowledge, persistence, and motivation to encourage the mentee to vicariously experience CR as desirable and attainable. There might, however, be competing mechanisms contributing to the positive effect in our findings, for example, different commuting distances in the two study groups. The distance from home to CR is an important factor affecting accessibility to health facilities (Mseke et al. 2024) and this is a known barrier to CR attendance (Pedersen et al. 2018b). Thus, the finding that some mentees were offered CR closer to home than controls might also partly explain the positive effect on CR attendance in the RCT (Pedersen et al. 2024). Our data suggested several barriers to lifestyle changes (diet and physical activity) for example, lack of perceived need for lifestyle changes, comorbidities, and the potentially negative impact on quality of life caused by radical lifestyle changes. These factors are in line with previous studies among older, vulnerable heart patients (Laursen et al. 2021; Pedersen et al. 2021), indicating that lifestyle changes are challenging within this group. Maintaining a good quality of life is pivotal in this group, as low quality of life is a predictor of mortality (Xu et al. 2023). Thus, rehabilitation programs and lifestyle modification programs must include the patient perspective in order to work. However, our limited results concerning dietary changes may also be attributed to the use of the HeartDiet food frequency questionnaire. A recent study has raised questions about the sustainability of this screening tool, as only a small number of individuals from the general population can achieve a heart‐healthy status (Kristensen et al. 2024).

Finally, our analysis illustrates the resilience displayed by some older mentees with considerable life experience. They showcase high levels of self‐efficacy, which may minimise the effect of mentor support on psychological outcomes such as anxiety and depression, as self‐efficacy is associated with lower levels of anxiety and depression (Thomet et al. 2018). This finding suggests that mentor support might be more effective in a younger IHD population, where IHD seems to have a greater negative impact on everyday life and mental wellbeing (Pedersen et al. 2021).

The findings related to the effect of mentor support on physical activity illustrated the importance of the mentor being a positive role model with experience‐based knowledge. As this provided authentic motivation, it was a positive mechanism, possibly affecting the physical activity of the mentee; however, only on a small scale, possibly due to the COVID‐19 situation at the time. Physical activity has a positive impact on improving health and quality of life among the elderly. Regular physical activity can contribute to reducing the risk of falls, maintaining good physical function, and maintaining a good quality of life (Ciumărnean et al. 2021). In the analysis of the primary RCT, we implemented a criterion for physical activity exceeding five sessions of 30 min per week, aligning with the World Health Organisation's recommendation of engaging in physical activity for more than 150 min per week (Gelius et al. 2020). Conversely, for the exploratory descriptive analysis, we adopted a higher threshold of physical activity surpassing six sessions per week, consistent with the prescribed level of physical activity advocated by Danish health authorities (Gelius et al. 2020). While the primary RCT utilised the lower threshold, deemed potentially more feasible in this population, the data suggest that the most notable changes occurred when employing the higher threshold. Consequently, the selected threshold will influence the outcomes, prompting consideration that exceeding six sessions of physical activity per week may be attainable in some older, vulnerable individuals.

## 12 Strengths and Limitations

This small sample process‐outcome evaluation identified facilitators and barriers that impact the peer‐mentor intervention beyond what an RCT can capture. The study occurred during the COVID‐19 pandemic, which may have affected mentor‐mentee interactions, participation rates, and consequently, study outcomes. Investigator triangulation enhanced the study's trustworthiness, with multiple researchers collaboratively analysing findings. The mixed methods design integrated established qualitative research approaches to enhance credibility, along with quantitative approaches using validated scales to rigorously evaluate quantitative study endpoints. Since interviews were conducted with participants who volunteered for a peer‐mentor intervention focused on CR attendance, there may be selection bias. Our informants might be more likely to be motivated for peer support and CR attendance and, thus, not fully representative of the post‐MI population. The study addressed issues related to legitimacy by integrating qualitative and quantitative findings through joint displays, enabling meta‐inferences. Specifically, the study focused on both integration legitimacy and weakness minimisation legitimacy by leveraging the strengths of qualitative and quantitative methods to achieve its research objectives (Younas et al. 2020). Our study was limited by reliance on volunteer in‐person resources to offer peer‐mentor support. In recent years, numerous web‐based interventions have emerged for the prevention and rehabilitation of patients with IHD. While these interventions might require less in‐person time, adherence might be lower in older, vulnerable patients (Sassone et al. 2024).

## 13 Conclusion

The study suggests several implications for practice, policy, and future research. Moreover, it offers compelling evidence for the effective mechanisms of peer‐support, indicating that peer‐mentor programs could be integrated with CR practice to improve patient engagement and attendance. However, it is essential to recognise both the resilience in this group and address barriers related to lifestyle changes. These factors may diminish the impact of mentor support, particularly among older individuals with ischaemic heart disease. It is critical to ensure that peer‐mentors possess the necessary qualifications for their role and provision of motivational, experience‐based support. However, it is also necessary to conduct a thoughtful assessment of whether the mentee population genuinely requests or requires mentor support, as this appears to be essential for optimising outcomes. To ensure that peer‐support is accessible to all patients in need, future research should aim to elucidate the specific mechanisms of mentor interventions across diverse populations, including younger individuals with IHD. Finally, effective implementation necessitates the allocation of policy awareness and resources to develop and sustain these mentor programs.

## Author Contributions

Made substantial contributions to conception and design, or acquisition of data, or analysis and interpretation of data; M.P., B.B.B., R.A.P., T.B., I.E. and D.O. Involved in drafting the manuscript or revising it critically for important intellectual content; M.P., B.B.B., R.A.P., T.B., I.E. and D.O. Given final approval of the version to be published. Each author should have participated sufficiently in the work to take public responsibility for appropriate portions of the content; M.P., B.B.B., R.A.P., T.B., I.E. and D.O. Agreed to be accountable for all aspects of the work in ensuring that questions related to the accuracy or integrity of any part of the work are appropriately investigated and resolved; M.P., B.B.B., R.A.P., T.B., I.E. and DO.

## Ethics Statement

The study conformed to the principles outlined in the Declaration of Helsinki. The Danish Scientific Ethical Committee (Journal‐nr.: H‐20038452) and the Danish Data Protection Agency (project‐number 19‐004) approved the study.

## Consent

Written informed consent was obtained from all mentees. Peer‐mentors signed a confidentiality agreement.

## Conflicts of Interest

The authors declare no conflicts of interest.

## Data Availability

The data that support the findings of this study are available from the corresponding author upon reasonable request.
